# Development and face validation of ultrasound-guided renal biopsy virtual trainer

**DOI:** 10.1049/htl.2019.0081

**Published:** 2019-11-26

**Authors:** Andinet Enquobahrie, Sam Horvath, Sreekanth Arikatla, Avi Rosenberg, Kevin Cleary, Karun Sharma

**Affiliations:** 1 Medical Computing, Kitware Inc, Carrboro, NC, USA; 2 School of Medicine, Johns Hopkins University, Baltimore, MD, USA; 3 Sheikh Zayed Institute for Pediatric Surgical Innovation, Children's National Health System, Washington, DC, USA

**Keywords:** biomedical ultrasonics, public domain software, paediatrics, computer based training, diseases, patient treatment, medical computing, surgery, biomedical education, kidney, needles, kidney failure, renal pathologies, competent biopsy technique, high yield biopsy samples, virtual simulator, procedural skill competence, US-guided renal biopsy, low-cost hardware components, open source software libraries, face validation, US images, needle visualisation, US-guided needle biopsy, clinical experts, clinical validation study, ultrasound-guided renal biopsy virtual trainer, chronic kidney disease, adult-paediatric nephrologists, interventional-diagnostic radiologists, hand-eye coordination, Web-based application, automated skill assessment, tracking modules, time 3.0 year to 23.0 year

## Abstract

The overall prevalence of chronic kidney disease in the general population is ∼14% with more than 661,000 Americans having a kidney failure. Ultrasound (US)-guided renal biopsy is a critically important tool in the evaluation and management of renal pathologies. This Letter presents KBVTrainer, a virtual simulator that the authors developed to train clinicians to improve procedural skill competence in US-guided renal biopsy. The simulator was built using low-cost hardware components and open source software libraries. They conducted a face validation study with five experts who were either adult/pediatric nephrologists or interventional/diagnostic radiologists. The trainer was rated very highly (>4.4) for the usefulness of the real US images (highest at 4.8), potential usefulness of the trainer in training for needle visualization, tracking, steadiness and hand-eye coordination, and overall promise of the trainer to be useful for training US-guided needle biopsies. The lowest score of 2.4 was received for the look and feel of the US probe and needle compared to clinical practice. The force feedback received a moderate score of 3.0. The clinical experts provided abundant verbal and written subjective feedback and were highly enthusiastic about using the trainer as a valuable tool for future trainees.

## 1 Introduction

**Fig. 1:**
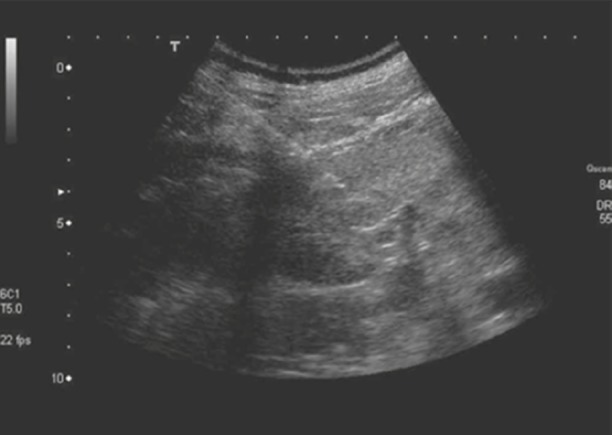
Needle pathway in kidney biopsy [4]

The overall prevalence of chronic kidney disease in the general population is ∼14% with more than 661,000 Americans having a kidney failure [1]. Ultrasound (US)-guided kidney biopsy is a critically important tool in the evaluation and management of renal pathologies. Although improvements in needle design and US imaging have been useful, excellent biopsy technique and skill are essential to consistently obtain high yield renal biopsy samples. Poor renal biopsy technique can potentially lead to significant complications and adverse lethal outcomes including severe bleeding, arteriovenous fistula and infection [2, 3]. Hence, technologies that improve the safety profile and accuracy of kidney biopsy are needed (Fig. 1).

Training in US-guided kidney biopsy procedures includes anatomical understanding, US image interpretation and eye-hand coordination required to successfully complete the procedure. Current biopsy training primarily relies on apprenticeship and simulation-based training using animal models and physical phantoms/mannequins. Apprenticeship learning requires the radiologist to show the technique to the trainee and guide him or her through the procedure. This can be a very time consuming and tedious process.

Simulation-based medical education removes the patient from the learning curve. Simulation plays an increasingly important part in the training and assessment of procedural skills because it allows for deliberate practice with opportunities for immediate feedback without risk to patient. It has been shown to be superior to traditional clinical medical education, and is recommended for achieving clinical skills [5, 6]. Over the past two decades, researchers have developed simulation prototypes that provide visual and haptic feedback with the goals of enhancing clinical training and medical education [7, 8]. Animal models are one training option with the advantages of being widely available, having a realistic feel of the tissues, and presence of blood vessels, bones and nerves and the ability to imbed targets in the model. These allow a realistic feel of tissue handling and US image acquisition for the learner. However, animal models have several disadvantages including high cost, the need for infection control, a limited shelf life of a few days, the need for refrigeration and the time needed to prepare the model with the targets.

**Fig. 2:**
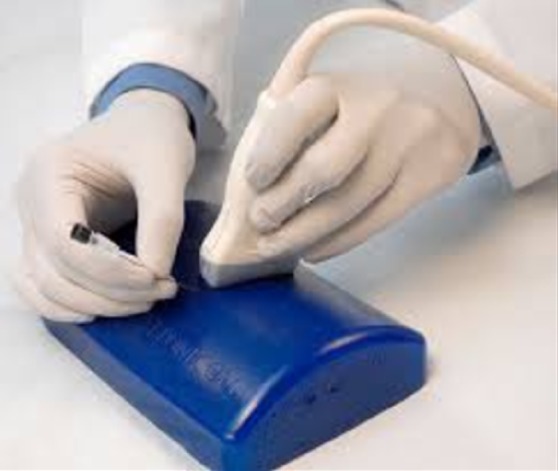
Blue phantom US biopsy trainer [9]

Physical phantoms are another training option. One popular product in the market is the Blue Phantom [9] (Fig. 2). The advantages of Blue Phantom include portability, a large scanning surface, long shelf life and reusability. The disadvantages include high cost, fixed targets with no ability to embed additional targets, visibility of prior needle tracks and non-tissue like force feedback. After wear from repeated usage, phantoms develop needle injection patterns which students can copy diminishing learning outcomes.

The development of a virtual renal biopsy simulator addresses the disadvantages of the animal models and phantoms. Touch of Life Technologies's OPUS Medical Skills Trainer [10] provides training for a variety of medical procedures, but with the anatomy from a limited collection of datasets, including the Visible Human. 3D Systems Simbionix U/S mentor [11] uses simulated US images from idealised anatomy, but does include different types of pathologies. These systems are all closed source (not easily extendable) and very expensive for wider adoption. These simulators do not offer a wide range of high-fidelity anatomies. Furthermore, in these types of simulators, users obtain valuable psychomotor skills but no rigorous education in the relevant US anatomy. Hence, there is a need for a cost-effective virtual simulator that will help nephrologists, radiologists and interventional radiologists train in the US imaging interpretation skills specific to performing a needle biopsy and learn manual dexterity in needle handling with the aid of tactile feedback. The technology built in this project has the potential to be used not only in renal biopsy but other clinical procedures such as liver biopsy (which has a higher volume of patients and therefore broader appeal to providers according to expert and user interviews), transjugular and laparoscopic renal biopsy (these procedures represent reliable alternatives to conventional percutaneous biopsy in patients at high risk of bleeding).

## 2 Methods

**Fig. 3:**
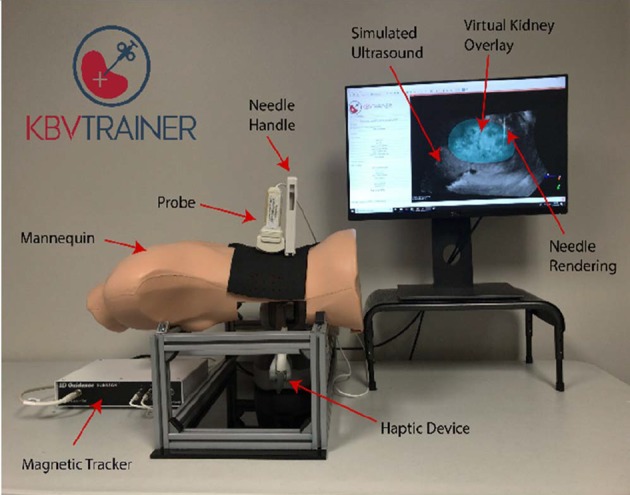
KBVTrainer setup showing the hardware and the software user interface

We developed a low-cost, virtual simulator called KBVTrainer (Kidney Biopsy Virtual Trainer), for US-guided kidney biopsy training. KBVTrainer is intended to train radiologists, nephrologists and interventional radiologists to improve procedural skill competence in US-guided renal biopsy. This advanced virtual simulator for real-time US-guided renal biopsy training provides several advantages including acceleration of the training of US-guided renal biopsy in a risk-free environment to improve the safety of kidney biopsy and ensuring that the biopsy procedure yields high-quality specimen. The assembled hardware for KBVTrainer is shown in Fig. 3. The simulator consists of (i) a mannequin, (ii) real US probe, (iii) real biopsy needle, (iv) electromagnetic tracker (Ascension 3D Guidance trakSTAR) to track the position and orientation of the US probe and (v) haptic device (3D Systems Geomagic Touch) for force feedback at the needle. Software visualisation and hardware interfacing for the trainer were provided by 3D Slicer [12] and PLUS Toolkit [13], respectively. 3D Slicer is an open source platform for medical image analysis and visualisation. It provides capabilities for image re-slicing, fiducial registration and many other functions for image-guided therapy. PLUS Toolkit is an open source package that specialises in hardware interfacing and data acquisition for medical imaging. PLUS manages the hardware interface for the Ascension 3DG device. To model needle to tissue interaction and interface with the Geomagic Touch device, we used Interactive Medical Simulation Toolkit (iMSTK), an open-source, interactive medical simulation toolkit designed for rapid prototyping of interactive simulation applications [14]. iMSTK provides an easy to use framework that can be extended and interfaced with other third-party libraries for the development of medical simulators without restrictive licenses. Use of these libraries as a platform allowed for rapid prototyping of the simulator.

**Fig. 4:**
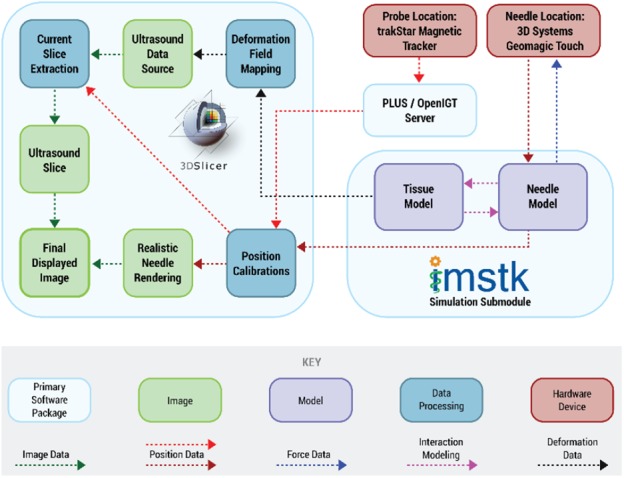
Workflow diagram showing the key processing units and the flow of image, position, force and deformation data

The workflow of the system is depicted in Fig. 4. The Slicer application receives tracking data of the US probe from a magnetically tracked sensor from PLUS over a local network connection. Needle tracking data is received directly through iMSTK. Using the probe's tracking information, the pre-acquired US volume is resliced along the plane of the probe in order to generate an image that reflects the current location and orientation of the probe with respect to the mannequin. A synthetic US image of the needle is generated using the needle's tracking data. This image is fused with the US image to simulate the real needle inside the tissue. The needle position and orientation data are used in the iMSTK submodule to drive the needle–tissue interaction model. Deformation data from the needle–tissue interaction will then be used to deform the displayed US volume, showing real-time ‘interaction’ with the image. The computed force data from the needle–tissue interaction model is transmitted back to the haptic device through iMSTK allowing the user to experience realistic tactile feedback. We used a mannequin to emulate the tactile feedback that trainees experience while scanning a real patient and to provide trainees with spatial awareness of the US scanning plane with respect to the patient's anatomy. Modules from the Slicer IGT extension were used to manage the registration and calibration of the coordinate systems of the hardware device, and to drive the re-slicing of the US volume to obtain the displayed image. In particular, the Volume Reslice Driver module allows for programmatically setting the orientation and location of a slice to be extracted from a volume. The extraction is performed by vtkImageReslice [15] class, by rotating, translating and resampling the volume so that it is axis-aligned with the desired slice.

To model the needle–tissue interaction, we first modelled the kidney deformation dynamics in 3D. For this purpose, we generated a 3D model using the pre-acquired 3D US volume. The kidney models were generated from a manual segmentation of the 3D US volume by an expert clinician. The surface mesh was generated using the Segmentations module in 3D Slicer. This module allows the import and export of segmentation data to several representations (label-map, contours and surface mesh). The labelmap to model conversion logic uses VTK's Flying Edges algorithm [16].

Next, the mesh is further processed by smoothing and decimation to obtain a useable surface mesh. TetGen [17] was used to perform Delaunay tetrahedralisation to generate a volumetric mesh representation from the surface. The surface mesh was further processed using repeated application of Quadratic edge collapse decimation and surface preserving Laplacian smoothing in Meshlab [18]. The resulting surface mesh was used as input for creating conformal tetrahedral mesh using TetGen resulting in a mesh of 2591 elements. The kidney volume is then modelled using co-rotational finite elements discretised in time using a backward Euler time stepping scheme. We use linear shape functions on the 3D tetrahedral elements to drive the finite-element formulation [19]. The needle is modelled as an idealised rigid straight line controlled by an external user through manipulation of the GeoMagic Touch haptic device. Such an idealisation is justified since the bending of the kidney biopsy needle observed during renal biopsy is limited. This assumption was supported by our clinical lead. For the tip of the needle to reach the target area inside the kidney, it needs to travel through various layers of the tissue (skin, subcutaneous fat, muscle, retroperitoneal fat etc.). Each tissue layer poses different levels of resistance to the movement of the needle as well as the forces perpendicular to the needle axis. The needle is modelled as an idealised rigid straight line controlled by an external user through manipulation of the haptic device. At each frame the nearest nodes to the needle within a certain threshold are computed by checking the shortest of the node to the needle axis. The kidney volume was modelled using co-rotational finite elements discretised in time using backward Euler time stepping scheme. Young's Modulus and Poisson's ratio of 0.1 MPa and 0.3 are used, respectively. Dirichlet boundary conditions are enforced on the nodes of the surface of the kidney corresponding to the renal pelvis area of the kidney using linear projection constraint framework described above.

In our preliminary study, we used a kidney mesh composed of 2591 tetrahedral elements. With this mesh model, the needle–tissue interaction simulation ran at 55–60 fps in iMSTK. We conducted a face validation study of our trainer at a research hospital. We acquired anonymised 3D US volume with good coverage of the kidneys. The US volumes were reconstructed using 3D Slicer. We invited five experts who were either adult/pediatric nephrologists or interventional/diagnostic radiologists to use and evaluate the simulator. All the experts had considerable experience performing US-guided needle biopsies within their specialty with experience ranging from 3 to 23 years. The study lasted ∼45 min for each participant and was conducted under an IRB approved protocol. Upon arrival, a brief overview of the clinical motivation and goals of the trainer and the current study was given by the study lead. The subjects were then asked to sign the informed consent form. They were also given a brief introduction to the project and demonstration of the simulator. After the demonstration, subjects were given the opportunity to use the trainer. The subjects were encouraged to test all the aspects of the simulator. Once the subjects had tested the simulator in its entirety, they were asked to complete a questionnaire which consisted of specific questions regarding various aspects of the simulator, as well as a section for subjective feedback. Additional verbal feedback received from the experts while performing on the trainer was recorded by the technical team.

The questionnaire consisted of seven questions probing various crucial aspects of the trainer such as overall appearance and usefulness of the US images, realism of the US probe and needle, usefulness of force feedback, potential usefulness of the trainer for overall US kidney biopsy and for specific skills required for biopsy. Specific questions include: (i) How realistic is the US appearance of the kidney on the trainer compared to clinical cases? (ii) How useful is it to have real US images in the trainer to guide US-guided kidney biopsy technique (versus a kidney model)? (iii) How realistic is US probe and needle handling (how it looks and feels), compared to clinical biopsy cases? (iv) How useful is the force feedback (sensation of resistance at the needle tip while advancing the needle into the kidney) aspect of the trainer? (v) Does this trainer show promise in being useful tool in learning a safe kidney biopsy technique? (vi) Please rate the potential usefulness of this kidney biopsy simulator in learning needle visualisation, tracking and steadiness skills. (vii) Please rate the potential usefulness of this kidney biopsy simulator in learning needle visualisation, tracking and steadiness skills. (viii) Please rate the potential usefulness of this kidney biopsy simulator in learning needle visualisation, tracking and steadiness skills. (ix) Did you encounter any confusion when you were using the *KBVTrainer*? If YES, what was the confusion? (x) Did you encounter any confusion when you were using the *KBVTrainer*? If YES, what was the confusion? (xi) How did you learn to do US-guided kidney biopsy? (xii) How could the simulator be used to help in the training process for current residents and fellows?

## 3 Results

**Fig. 5:**
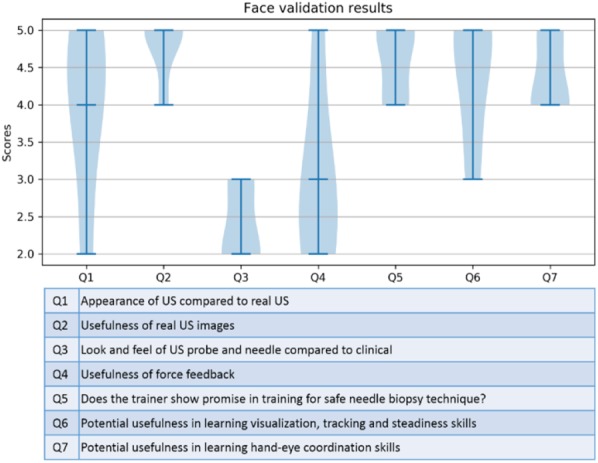
Chart summarising the scores obtained for various questions related to different aspects of the KBVTrainer

The subjects were asked to rate these aspects on a 5-point Likert scale with 1 being ‘not useful/unrealistic’ to 5 ‘useful/very realistic’. Fig. 5 shows the scores from each subject. The trainer was rated very highly (>4.4) for the usefulness of the real US images (highest at 4.8), potential usefulness of the trainer in training for needle visualisation, tracking, steadiness and hand-eye coordination, and overall promise of the trainer to be useful for training US-guided needle biopsies. The lowest score was received for the look and feel of the US probe and needle compared to clinical at 2.4. The force feedback received a moderate score of 3.0. The experts showed considerable enthusiasm in using the trainer which is evident from the abundant verbal and written subjective feedback provided. Some experts felt that the quality of the US is lower compared to that observed in the clinic and therefore had difficulty locating the boundaries of the kidney during the training. More experienced subjects were however fine with the quality.

The experts also noted that the needle is not as constrained compared to real renal biopsy understandably due to not modelling the extraneous tissue that occupies the space between the skin surface and that of the kidney. One expert noted that they would like to feel the puncture force at the time of piercing the kidney surface. Some experts wanted a Doppler overlay to help locate the blood vessels stating that it would be useful for the novice.

## 4 Discussion and conclusion

In this work, we developed KBVTrainer to address the limitations in existing US-guided biopsy simulators. KBVTrainer is a virtual simulator that provides a cost-effective, high-fidelity environment for US-guided kidney needle biopsy training. The key contributions of this work are as follows. (i) Use of real patient images: for trainees to grasp the complexity and challenge of interpreting kidney anatomy with US, the simulator used high-quality 3D kidney US images from real patients. (ii) High fidelity needle–tissue interaction modelling algorithms: we incorporated a haptic device for needle insertion to provide realistic simulation of the force profile that a clinician would experience during the insertion of a needle through the various tissue layers surrounding the kidney. We developed advanced techniques (a) to model and simulate the deformations of various tissue layers that are coupled with the needle motion, (b) to estimate the frictional forces between the walls of the needle with the tissue surrounding it and (c) to estimate puncture forces given the shape of the needle tip. (iii) An affordable and easily extendable simulator: we built a low-cost simulator using off-the-shelf hardware components and powerful open source visualisation and interactive simulation libraries. Three aspects of KBVTrainer lend to its extendable nature (i) modular framework employed using 3D Slicer and iMSTK (ii) generic needle–tissue interaction algorithms suitable for extension to other biopsies and (iii) generic hardware design.

The current deformation dynamics ran at 55–60 frames per second (fps) while generating forces. These forces are used asynchronously by the haptics thread that runs at up to 1000 fps. A zeroth order hold is enforced until the new force data is furnished by the simulation frame. This latency can cause some discontinuity in the forces. This can be improved by (i) improving the frame rate of the simulation thread, (ii) applying moving average smoothing filter to help reduce sharp changes in the forces.

In conclusion, the face validation conducted provided an understanding of the quantitative challenges and opportunities in virtual simulator for kidney biopsy. As part of the future work, we will improve the technology based on clinical and user feedback, develop automated skill assessment and tracking modules deployable in a web-based application and conduct a clinical validation study.
